# Dimorphism in Fungal Pathogens of Mammals, Plants, and Insects

**DOI:** 10.1371/journal.ppat.1004608

**Published:** 2015-02-12

**Authors:** Gregory M. Gauthier

**Affiliations:** Department of Medicine, University of Wisconsin, Madison, Wisconsin, United States of America; The University of North Carolina at Chapel Hill, UNITED STATES

## Introduction

The capacity for pathogenic fungi to change morphology during their lifecycle is widespread. However, relatively few fungi are considered dimorphic, which refers to the ability to switch between two morphologies, yeast and hyphae (Table 1). These pathogens can be roughly subdivided into thermal (morphologic switch induced by temperature) and non-thermal dimorphic fungi (Table 1).

**Table 1:** Dimorphic fungal pathogens. *

Fungus	Phylum	Disease	Major stimuli for the morphologic switch
**Mammalian pathogens**			
*Blastomyces dermatitidis* ^†^ ^,^ ^‡^	Ascomycota	Blastomycosis	Temperature
*Coccidioides immitis* ^†^ & *posadasii* ^†^	Ascomycota	Coccidioidomycosis	Temperature
*Histoplasma capsulatum* ^†^	Ascomycota	Histoplasmosis	Temperature
*Emmonsia* spp. ^†^	Ascomycota	Emmonsiosis	Temperature
*Lacazia loboi* ^†^ ^,^ ^§^	Ascomycota	Lacaziosis	Temperature
*Penicillium marneffei* ^†^ ^,^ ^¶^	Ascomycota	Penicilliosis	Temperature
*Paracoccidioides brasiliensis* ^†^ & *lutzii* ^†^	Ascomycota	Paracoccidioidomycosis	Temperature
*Sporothrix schenckii* ^†^	Ascomycota	Sporotrichosis	Temperature
*Hortaea werneckii*	Ascomycota	Tinea nigra	Temperature, nutrition, inoculum size
*Malassezia furfur*	Basidiomycota	Pityriasis versicolor	L-DOPA
*Mucor* spp.	Mucoromycotina^#^	Mucormycosis	O_2_ & CO_2_ tension
*Cokeromyces recurvatus*	Mucoromycotina^#^	Mucormycosis	Temperature, Nutrients, O_2_ & CO_2_ tension
			
**Plant pathogens**			
*Ophiostoma ulmi* & *O. novo-ulmi*	Ascomycota	Dutch elm disease	Nitrogen source, quorum sensing
*Holleya sinecauda*	Ascomycota	Mustard seed rot	Unknown
*Taphrina deformans*	Ascomycota	Peach & almond leaf curl	Unknown
*Ustilago maydis*	Basidiomycota	Corn smut	Pheromones, plant lipids, plant hydrophobicity, pH, nitrogen
			
**Insect Pathogens**			
*Beauveria bassinia*	Ascomycota	White muscardinePlant endophyte	Insect hemolymph
*Metarhizium* spp.	Ascomycota	Green muscardinePlant endophyte	Insect hemolymph
*Ophiocordyceps unilateralis*	Ascomycota	“Zombie ant”	Insect hemolymph

*This table includes the most common dimorphic fungal pathogens of mammals, plants, and insects; thus, it is not all-inclusive. *Saccharomyces cerevisiae*, which rarely causes human infection, is not included because it converts between yeast and pseudohyphae. Although *Cryptococcus neoformans* converts to a filamentous form during mating, is not traditionally considered dimorphic fungus. *Candida albicans* can be considered a polymorphic fungus because it grows as yeast, pseudohyphae, and hyphae.

† Thermally dimorphic fungi.

‡ Phylogenetic analysis suggests that *Blastomyces* may include two species, *B. dermatitidis* and *B. gilchristii* sp. nov.

§ Although *Lacazia loboi* cannot be grown in vitro, it is phylogenetically related to *P. brasiliensis* and other thermally dimorphic fungi.

¶ *Penicillium marneffei* has been renamed *Talaromyces marneffei*.

# The taxonomic location of the subphylum mucoromycotina is uncertain (the zygomycota phylum is obsolete).

Worldwide, the thermally dimorphic fungi cause several million human infections each year. In the United States, *Histoplasma capsulatum* and *Coccidioides* spp. are estimated to infect 500,000 and 150,000 persons annually, respectively [1,2]. The incidence of coccidioidomycosis has recently increased and its endemic range has extended beyond the Southwest to include eastern Washington state [3]. Although the thermally dimorphic fungi typically infect healthy hosts, these pathogens account for 5.3% of fungal infections in solid organ transplant recipients [4]. Immunosuppressed patients are at risk for respiratory failure and extrapulmonary dissemination [4].

Phytopathogenic dimorphic fungi have had a major impact on urban landscapes and agriculture. *Ophiostoma ulmi*, which caused the first Dutch elm disease epidemic, has been replaced by a more virulent species, *Ophiostoma novo-ulmi*, which has destroyed millions of elm trees in the US and Europe [5]. *Taphrina deformans*, the etiologic agent of peach leaf curl, results in economic losses of $2.5–3 million in the US [6]. Although *Ustilago maydis*, which causes corn smut, is not a major agricultural threat, the galls (i.e., huitlacoche) caused by infection are eaten as a delicacy [7]. Entomopathogenic fungi have been utilized to control insects harmful to agriculture and to study how pathogens control host behavior [8,9].

## The Dimorphic Transition

The morphologic shift between hyphae and yeast is critical for the pathogenesis, virulence, and lifecycle of the dimorphic fungi. In the soil (22–25°C), the thermally dimorphic fungi grow as mycelia that produce infectious conidia (i.e., spores). Following soil disruption, aerosolized conidia and hyphal fragments inhaled into the lungs of a mammalian host (37°C) convert to yeast (or spherules for *Coccidioides*) to cause pneumonia. Although temperature is the major stimulus, other factors such as CO_2_, cysteine, and estradiol influence conversion and growth at 37°C [10]. Binding of estradiol to cell surface receptors accelerates the growth of *Coccidioides* spherules and inhibits the phase transition of *Paracoccidioides brasiliensis* at 37°C [10]. This may explain why pregnant women are at risk for disseminated coccidioidomycosis and why paracoccidioidomycosis rarely infects women [10].

Varied stimuli influence the morphologic switch for non-thermally dimorphic fungi. *Mucor* spp. grow as yeast under anaerobic conditions and hyphae when oxygen is present (e.g., tissue) [11]. In contrast, *Cokeromyces recurvatus* grows as budding yeast in humans and has been misidentified as *Paracoccidioides* [12]. *Malassezia furfur*, the cause of pityriasis versicolor, converts from yeast to hyphae in the presence of L-DOPA [13]. *Hortaea werneckii*, which serves as a model for halotolerance, grows as yeast at ambient temperature and converts to hyphae to cause black skin discoloration known as tinea nigra [14]. Mating pheromones are critical for initiating the hyphal transition in *U. maydis*, but are dispensable for *T. deformans* [6,7]. Nitrogen sources, lipoxygenases, cyclooxygenases, and quorum sensing molecules contribute to yeast-mycelial dimorphism in *Ophiostoma* spp. [5,15]. The in insecta stimuli that promote yeast-like growth for entomopathogenic fungi are poorly understood; however, cultivation in submerged cultures induces yeast-like development [8].

## How Does the Morphologic Switch to Yeast Impact Pathogenesis?

To cause invasive infection in humans (e.g., pneumonia), fungi must overcome structural, thermal, and immunologic barriers. Intact skin is resistant to invasive infection and cutaneous pathogens such as *M. furfur* and *H. werneckii* only penetrate the superficial epidermal layers by growth as hyphae, not yeast [14]. The small-sized conidia of the thermally dimorphic fungi can bypass structural lung defenses (e.g., ciliated epithelia) and enter the lower respiratory tract. Once in the lung, conidia bind to innate immune cells via lectin and mannose receptors, become phagocytozed, germinate, and replicate intracellularly as budding yeast [16,17]. Preformed transcripts may facilitate rapid adaption of conidia that germinate as yeast to the host environment. *H. capsulatum* conidia are enriched for transcripts involved with stress resistance, DNA replication, cell signaling, and transcriptional regulation (e.g., *RYP1*) [16].

During the past decade, there have been substantial advances in understanding the transition to yeast. In *Blastomyces dermatitidis* and *H. capsulatum*, a hybrid histidine kinase encoded by *DRK1* (dimorphism-regulating kinase-1) is important for the transition to yeast at 37°C and virulence [18]. Deletion of *DRK1* results in *B. dermatitidis* and *H. capsulatum* cells that grow as hyphae (instead of yeast) at 37°C and renders these pathogens avirulent [18]. This provided genetic proof that the transition from hyphae to yeast is essential for virulence [18]. Moreover, a homolog of *DRK1* in *Penicillium marneffei, DRKA*, is critical for germinating conidia to develop into yeast within macrophages at 37°C [17]. In addition to *DRK1*, transcription factors *RYP1–4* (required for yeast phase) in *H. capsulatum* promote the transition to yeast [19–21]. Knockdown of *RYP1–4* results in hyphal growth at 37°C and a failure to up-regulate yeast-phase genes critical for virulence [19–21]. The transcriptional profile of *RYP1–4* knockdown strains at 37°C is similar to wild-type *H. capsulatum* mycelia [21]. Moreover, RYP1–3 physically interact and bind DNA to regulate a network of genes involved with temperature adaptation [21].

During the transition to yeast, genes critical for immune evasion, intracellular survival, and dissemination are up-regulated. Many of these genes, including *BAD1, SOWgp, CBP1*, and *YPS3*, are transcribed only in the yeast phase. *B. dermatitidis* BAD1 (formerly WI-1) is an adhesin that attaches yeast to host cells by binding heparan sulfate, CR3, and CD14 [22]. BAD1 promotes immune evasion by blocking TNF-α production and inhibiting CD4+ T lymphocyte activation [22]. *Coccidioides* spherule outerwall glycoprotein (SOWgp) and *P. brasiliensis* glycoprotein 43 (gp43) facilitate adhesion of yeast to extracellular matrix proteins and contribute to virulence [23,24]. In *H. capsulatum*, alpha-(1,3)-glucan, which contributes to virulence in chemotype II strains, is located in the outermost layer of the cell wall, and blocks β-glucan recognition by immune cells [25]. *H. capsulatum* CBP1 (calcium binding protein-1) is a secreted, saposin-like protein that binds calcium (and possibly lipids) and is essential for survival within macrophages [26]. YPS3 (yeast-phase specific-3) is a secreted protein that promotes dissemination of *H. capsulatum* to the liver and spleen [1]. Deletion of *BAD1* and *CBP1* renders yeast avirulent without affecting the phase transition [22,26].

Insect and plant pathogenic fungi must also penetrate structural defenses and avoid immune defenses to cause invasive disease. Once inside insect hemocele, the invading filamentous form switches to yeast-like growth. These yeast-like cells, which are also known as blastospores, replicate by budding, evade host immune defenses, promote dissemination, and alter host behavior. The MAD1 adhesion in *Metarhizium robertsii* promotes conidial adherence and blastospore formation [27]. *MAD1* deletion reduces blastospore production, which is associated with attenuated virulence [27]. *Beauveria bassiana* blastospores shed cell surface carbohydrates to avoid detection by immune defenses [8]. In *Ophiocordyceps unilateralis s.l*. (“zombie-ant fungus”), yeast-like cells invade the muscles and possibly the central nervous system and secrete toxic metabolites to alter the behavior of the infected *Camponotus* and *Polyrhachis* ants [9, 28]. Infected ants leave the colony and attach themselves via a “death grip” bite to a leaf or twig where they succumb to infection [9,28].

The importance of the yeast phase for plant pathogens is species specific. In *U. maydis*, diploid spores on the plant surface germinate to form haploid, budding yeast [7]. Yeast cells with opposite mating-type loci (i.e., *a* and *b* mating-type loci) fuse to form a filamentous dikaryon that penetrates the plant cuticle and invades tissue [7]. *T. deformans* also switches from budding yeast on the leaf surface to filamentous growth to invade plant tissue; however, mating is not required for the dimorphic switch [6]. For *O. ulmi* and *O. novo-ulmi*, yeast-like blastospores disseminate in the tree using the xylem network, whereas hyphae facilitate spread between vascular structures [5].

## How Do Hyphae Contribute to Pathogenesis?

Hyphal growth coupled with conidiogenesis promotes environmental survival, transmission to new hosts, and genetic diversity via mating. The ability of *Coccidioides posadasii* to infect healthy hosts and survive in the soil has contributed to long-range geographic dispersal [2,29]. Analysis of microsatellite loci from 163 *Coccidioides* isolates indicated that South American *C. posadasii* strains originated in Texas, US [29]. The southward migration of *C. posadasii* corresponded with human migration (and possibly other mammals) into South America [29]. Following host death, spherules convert to hyphae that grow in the carcass and surrounding soil [2].

For the thermally dimorphic fungi, sexual reproduction occurs when hyphae with opposite mating-type loci (e.g., MAT1–1 and MAT1–2) fuse and form cleistothecia, specialized structures that produce spores by meiosis [30]. *B. dermatitidis* and *H. capsulatum* with either mating-type loci can infect humans, but how mating contributes to virulence for these fungi is speculative [30]. Introgression of *C. posadasii* genes to *Coccidioides immitis* includes genes that are postulated to facilitate immune evasion [31]. For *O. novo-ulmi*, introgression of *O. ulmi* MAT1 and vegetative incompatibility genes has allowed the pandemic strain to mate, which, in turn, eliminates d-factor viruses (infected *O. novo-ulmi* have reduced virulence and survival) [32].

Several advances have illuminated the molecular biology underlying the yeast-to-hyphal transition. *B. dermatitidis SREB* and *H. capsulatum SRE1* homologs encode GATA transcription factors that regulate siderophore-mediated iron assimilation and the temperature-dependent conversion to mold [33,34]. *SREB* null mutants and *SRE1* knockdown strains fail to complete the conversion from yeast to mold at 22–25°C independent of exogenous iron concentrations [33,34]. A homolog of *SREB* and *SRE1* in *Cryptococcus neoformans, CIR1*, regulates iron uptake and thermotolerance [35]. This suggests a conserved role for GATA transcription factors in temperature adaptation. In addition to *SRE1*, a vacuolar ATPase, VMA1, involved with iron homeostasis in *H. capsulatum*, affects the mycelial transition at ambient temperature [36]. In *Penicillium marneffeii, HGRA* (hyphal growth regulator) and *TUPA* facilitate conversion and maintenance of mycelial growth at 25°C, respectively [37,38]. The rate of *H. capsulatum* and *B. dermatitidis* mycelial conversion is accelerated by N-acetylglucosamine (GlcNAc), which is mediated by *NGT1* and *NGT2* transmembrane transporters [39]. Collectively, these data suggest specific genes are involved with the initiation, kinetics, and maintenance of the morphologic switch.

For non-thermally dimorphic fungi, mycelial growth is important for superficial mycoses (e.g., pitryiasis versicolor), invasive mucormycosis, plant infections, and exit of entomopathogens from their insect host. In *Mucor circinelloides*, an agent of mucormycosis, the calcineurin regulatory unit encoded by *CNBR* is essential for the transition to pathogenic mycelia [11]. *CNBR* null mutants are locked in the yeast phase and have attenuated virulence [11]. Similarly, exposure of *M. circinelloides* to FK506, a calcineurin inhibitor, results in yeast growth; this may explain why transplant recipients treated with FK506 have lower rates of mucormycosis [11]. The mycelial transition in *U. maydis* has been extensively investigated and involves the interaction of two homeodomain transcription factors (bW, bE) along with MAPK and cAMP-PKA signaling cascades [7]. Once inside the plant, *U. maydis* proliferates, secretes proteins to suppress host defenses, and induces tumor (gall) growth [7]. The genes that regulate the blastospore-to-hyphal transition in entomopathogens, which is important for exiting the insect host and forming spores to transmit new infection, are poorly defined.

## Conclusions

The morphologic switch is essential for the pathogenesis of dimorphic fungi. For mammalian and insect fungi, the transition to a yeast or yeast-like growth results in altered cell wall composition as well as production of proteins to evade immune defenses or toxins to alter host behavior. Growth as yeast coupled with thermotolerance allows for replication within mammalian phagocytic cells, which promotes dissemination. For phytopathogens, the role of the yeast phase is species specific; it can be involved with sexual reproduction or dissemination within vascular tissue. Growth as mycelia promotes transmission to new hosts via conidia, and for the thermally dimorphic fungi has contributed to long-range geographic dispersal. Sexual reproduction occurs in the mycelial phase for most dimorphic fungi and has facilitated gene introgression to enhance survival and virulence. For non-thermally dimorphic fungi, mycelial growth is essential for invasive disease. Although a number of genes that govern the phase transition are known, how these genes fit into a larger network of regulated genes remains to be fully answered.
